# Congenital lumbar spinal stenosis with ossification of the ligamentum flavum in achondroplasia: a case report

**DOI:** 10.1186/1752-1947-8-88

**Published:** 2014-03-05

**Authors:** Kimio Saito, Naohisa Miyakoshi, Michio Hongo, Yuji Kasukawa, Yoshinori Ishikawa, Yoichi Shimada

**Affiliations:** 1 Department of Orthopedic Surgery, Akita University Graduate School of Medicine, 1-1-1, Hondo, Akita 010-8543, Japan

**Keywords:** Achondroplasia, Lumbar spinal stenosis, Ossification of the ligamentum flavum

## Abstract

**Introduction:**

Achondroplasia is a genetic disorder of bone growth. Congenital spinal stenosis is a well-known complication of this disease, but, to the best of our knowledge, no cases involving combined stenosis with congenital lumbar spinal stenosis and ossification of the ligamentum flavum in achondroplasia have been reported previously. In this report, we describe a case of a patient with congenital spinal stenosis with achondroplasia combined with ossification of the ligamentum flavum at the lumbar spine, which we treated with decompressive surgery.

**Case presentation:**

A 75-year-old Japanese woman with achondroplasia was unable to walk due to a neurological deficit of the lower extremities caused by congenital spinal stenosis that resulted from achondroplasia and ossification of the ligamentum flavum at the lumbar spine. Congenital spinal stenosis was observed from L1 to L5, and ossification of the ligamentum flavum was identified from L1/2 to L3/4. A decompressive laminectomy from L1 to L5 and removal of the ossification of the ligamentum flavum were performed. The patient’s neurological symptoms improved after surgery. She could walk with T-cane at the time of her four-year follow-up examination.

**Conclusion:**

In this report, we describe what is, to the best of our knowledge, the first known published case of ossification of the ligamentum flavum in congenital spinal stenosis associated with achondroplasia at the lumbar spine. Although resection of the ossification of the ligamentum flavum at the congenital spinal stenosis at the lumbar spine was technically difficult because of congenital narrowing of the spinal canal, thickening of the lamina and adhesion of the ossified ligamentum flavum, a wide laminectomy and resection of the ossification of the ligamentum flavum resulted in acceptable improvement of the patient’s neurological symptoms.

## Introduction

Achondroplasia is a genetic disorder of bone growth. The common features include short stature with disproportionately short limbs and a characteristic appearance of the head and face. Congenital spinal stenosis is a well-known complication of this disease [1]. Worsening of stenosis is usually due to degenerative changes of the spine, such as disc herniation, degenerative spondylosis and arthrosis [2]. However, symptomatic spinal stenosis caused by ossification of the ligamentum flavum (OLF) in achondroplasia has been previously reported in only one case at the thoracic spine [3]. In this report, we describe what is, to the best of our knowledge, the first published case of extremely rare congenital spinal stenosis caused by achondroplasia combined with OLF at the lumbar spine in an elderly woman.

## Case presentation

A 75-year-old Japanese woman presented with pain and weakness in her left leg. She had had low back pain for 10 years before she presented at our institution. She had gradually become unable to walk owing to impairment of the cauda equina or to maintain a sitting position because of increased pain in both legs, and for those reasons she was admitted to our hospital. She was short in stature, with a height of 118cm; she had an overly prominent forehead; and the bridge of her nose was scooped out. These features were considered consistent with achondroplasia.

**Figure 1:**
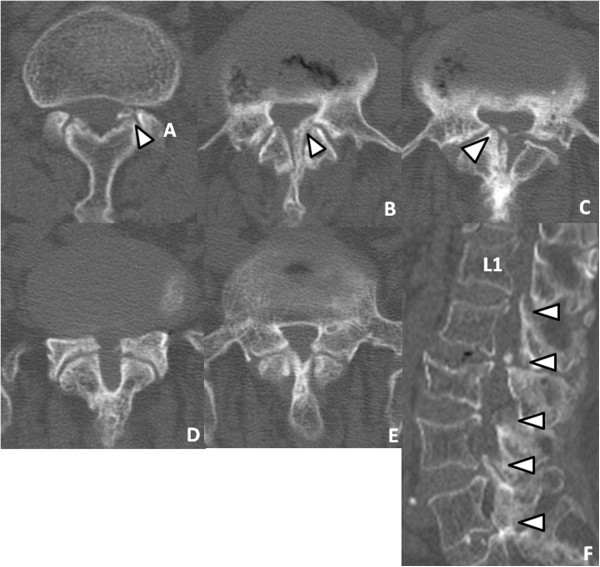
**Preoperative computed tomography. (A)** through **(E)** Preoperative computed tomographic scans show congenital spinal stenosis from L1 to L5, respectively. Axial images show ossification of the ligamentum flavum (OLF) at L1/2 **(A)**, L2/3 **(B)** and L3/4 **(C)** (arrowheads). **(F)** Sagittal scan shows OLF at L1/2 **(A)** to L3/4 **(C)** and spinal stenosis with thickened lamina and narrowing of the lateral recess at L4/5 and L5/S (arrowheads).

**Figure 2:**
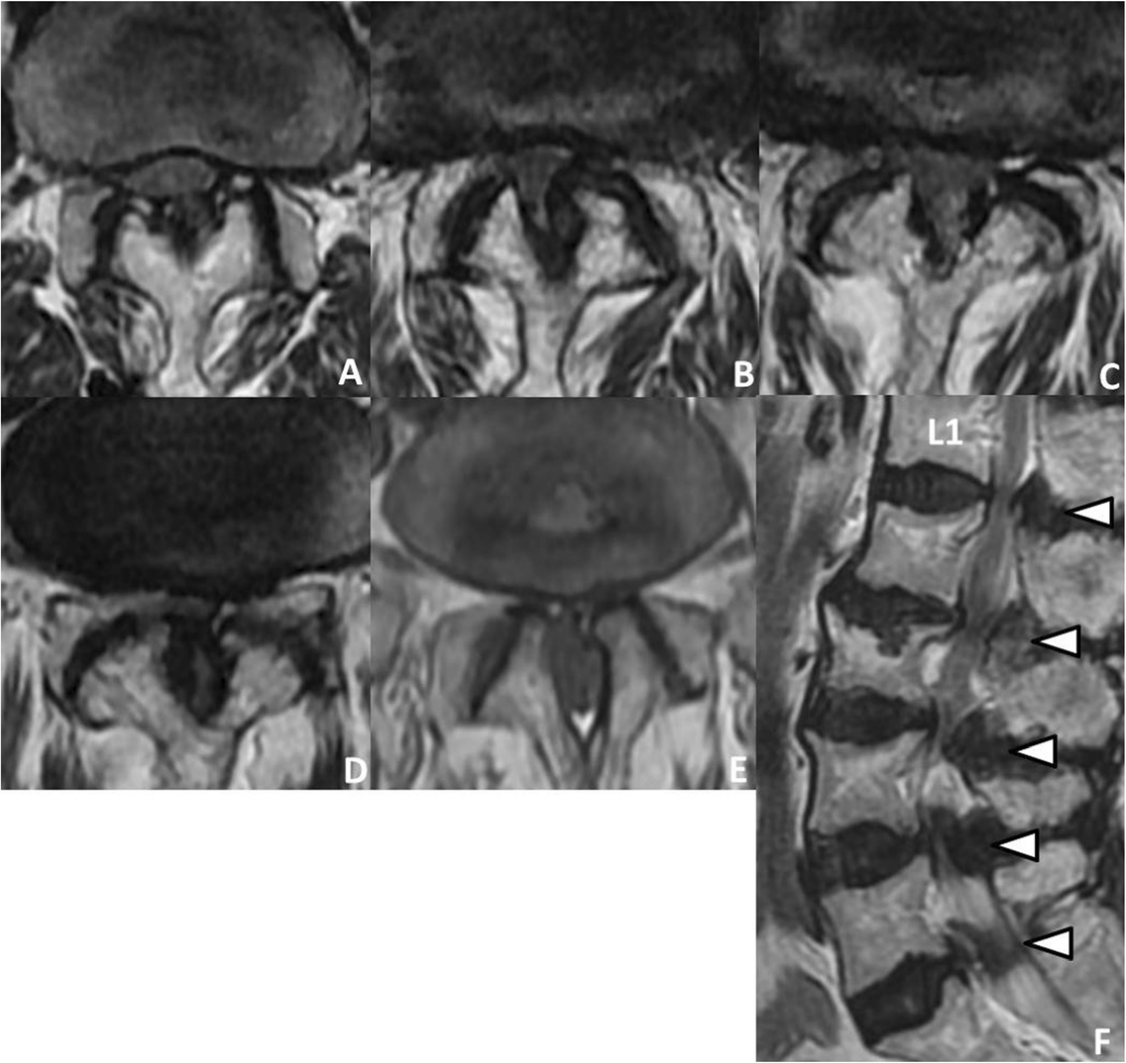
**Preoperative magnetic resonance images. (A)** through **(E)** Preoperative magnetic resonance imaging scans show extensive spinal stenosis from L1/2 **(A)**, L2/3 **(B)**, L3/4 **(C)**, L4/5 **(D)** and L5/S1 **(E)**. **(F)** Sagittal T2-weighted image reveals multiple stenosis at L1/2-L5/S (arrowheads).

**Figure 3:**
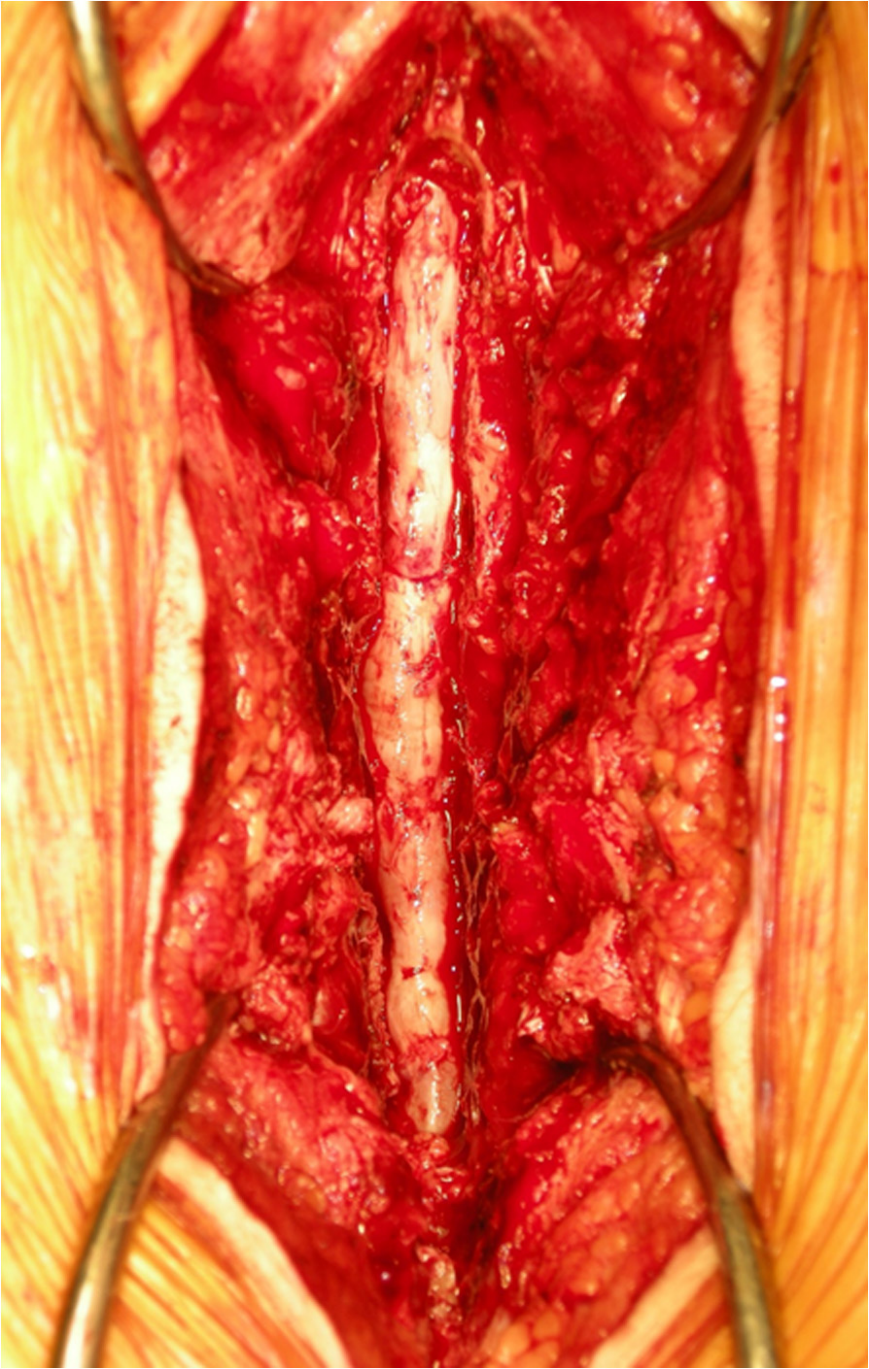
**Perioperative findings.** Laminectomy of L1 to L5 was performed to decompress the congenital spinal stenosis and to remove the ossification of the ligamentum flavum.

**Figure 4:**
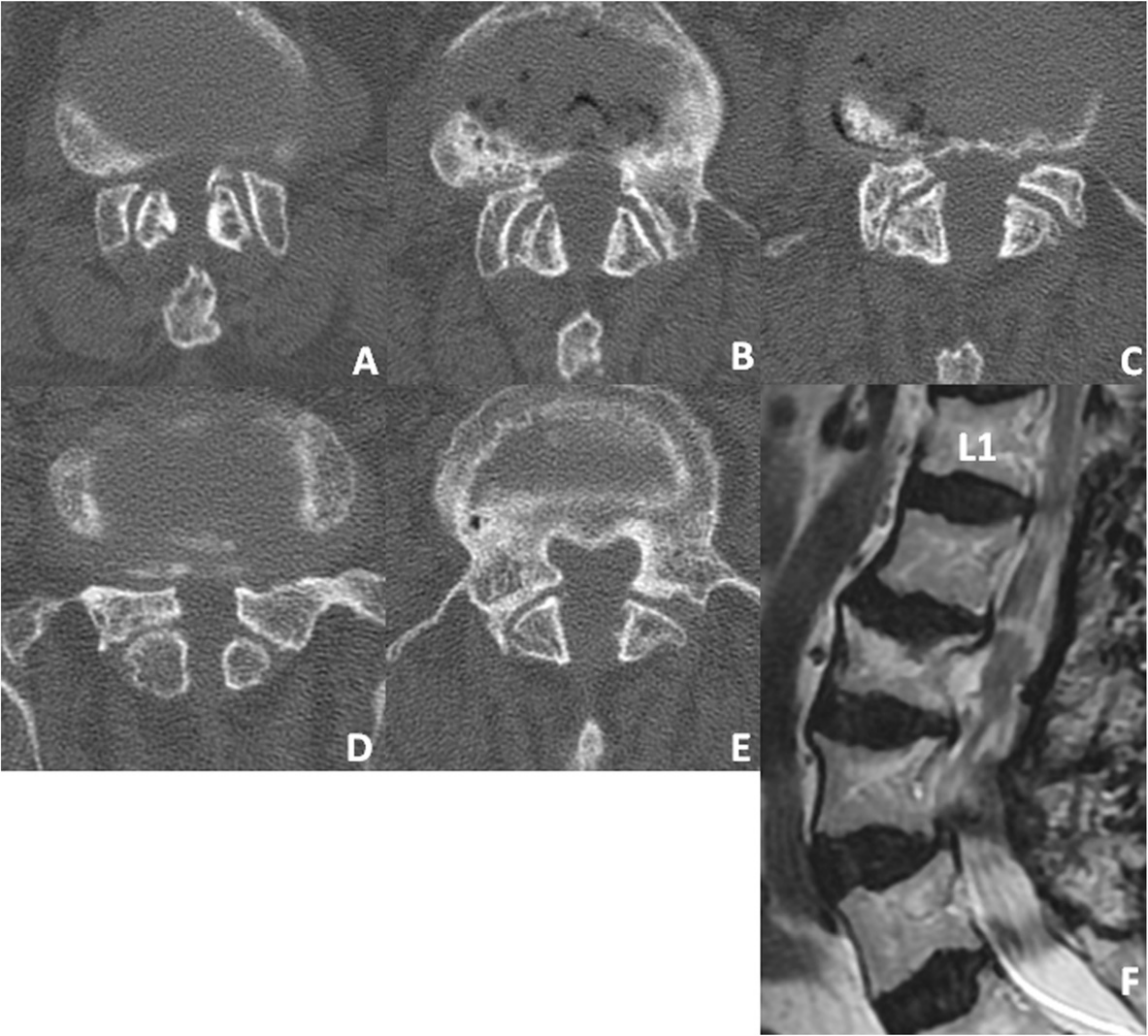
**Postoperative computed tomographic and magnetic resonance images. (A)** through **(E)** Computed tomographic scans show sufficient spinal canal enlargement at L1/2 **(A)**, L2/3 **(B)**, L3/4 **(C)**, L4/5 **(D)** and L5/S **(E)** achieved by performing laminectomies with partial resection of the OLF at L1/2 and total resection of the OLF at L2/3 and L3/4. **(F)** Postoperative sagittal T2-weighted magnetic resonance imaging scan reveals good decompression of the dural sac from L1/2 to L4/5.

Her neurological examination revealed weakness of the iliopsoas, tibialis anterior, extensor hallucis longus and flexor hallucis longus muscles, with grades of 4/5 recorded upon manual muscle testing. Her deep tendon reflexes were hypoactive in her bilateral lower extremities. She had sensory loss in the bilateral lower extremities and the perineal region. She also complained of mild bladder dysfunction.Axial computed tomographic (CT) scans revealed a congenital developmental deformity with spinal canal stenosis from L1 to L5 (Figure 1) and OLF at L1/2 (Figure 1A), L2/3 (Figure 1B) and L3/4 (Figure 1C). Interpedicular distances were narrow, especially at L4 and L5. Magnetic resonance imaging (MRI) showed narrowing of the spinal canal from L1/2 to L5/S1 (Figure 2). On the basis of these findings, we diagnosed the main cause of her symptoms as severe congenital spinal stenosis combined with OLF. Therefore, we performed a wide decompressive laminectomy and resection of the OLF from L1 to L5 using the spinous process-splitting approach. The spinous processes and laminae from L3 to L5 were in close contact with each other because of hyperlordosis at the lower lumbar spine. The ossified ligamentum flavum was closely adherent to the dura mater from L1/2 to L3/4. After the laminectomy and OLF resection, pulsation of the dura matter was observed (Figure 3). The patient’s neurological symptoms improved markedly after surgery. Postoperative CT and MRI scans revealed posterior decompression at the site of the resection of the laminae and OLFs from L1/2 through L5/S (Figure 4). The lateral portion of the OLF at the level of L1/2 remained, but sufficient decompression of the spinal canal was carried out from L2 through L5. The patient could walk with a T-cane one year after surgery and was still able to walk at the time of her four-year follow-up examination.

## Discussion

Achondroplasia is an autosomal dominant disorder that results from a characteristic mutation in the gene encoding fibroblast growth factor receptor 3 [4]. Achondroplasia is characterized by rhizomelic shortness of the limbs due to abnormalities of endochondral ossification. Spinal stenosis, which was classified as congenital developmental stenosis by Arnoldi *et al*. [5], is commonly identified. The achondroplastic spinal canal is one-third to one-half the size of a normal spine, secondary to decreased interpedicular distance caudally as well as shortened, thickened pedicles and laminae [2,6]. Nelson found that approximately 50% of patients with achondroplasia had neurological complications and that the onset of symptoms was common in patients from 30 to 50 years of age [7]. The neurological impairment was reported to be caused by several factors, including disc degeneration, kyphosis of the lumbar vertebrae, hypertrophy of the ligamentum flavum, bony spurs and thickened laminae and facet joints [8]. However, OLF has been reported as a cause of symptomatic spinal stenosis caused by achondroplasia in the thoracic spine in only one case [3]. Suzuki *et al*. [3] reported a case of spinal stenosis caused by achondroplasia with OLF observed at T4/5 and T9 to T12 compressing the thecal sac. OLF is a well-known cause of progressive thoracic myelopathy reported mainly in Asian patients [9,10]. The most common site of OLF requiring surgery is the thoracic spine [11]. The incidence of OLF at the lumbar spine has been reported to range from 8.6% to 11.3% [12,13]. A search of the literature identified no reports of OLF in the lumbar spine associated with achondroplasia. To the best of our knowledge, our present case is the first documented report of symptomatic OLF at the site of congenital lumbar spinal stenosis in achondroplasia.

The age at onset in the present case seemed to be older than that in common achondroplasia. The symptoms of lumbar spinal stenosis with achondroplasia usually occur earlier than those without achondroplasia. Although onset depends on the severity of developmental stenosis, age-related degeneration is also involved. In this regard, in our patient, the underlying developmental stenosis was likely not the sole cause of her symptoms, as it was not critical when she was young. Thus, the development of OLF with aging accelerated the severity of spinal canal stenosis, resulting in progressive symptoms requiring surgery at an older age.

Concerning treatment after diagnosis, it is important to decide the optimal timing of surgical intervention for achondroplastic patients with spinal stenosis. In the present case, decompressive wide laminectomy and resection of OLF were performed. Although a wide laminectomy was needed for resection of the adherent OLF, it was technically difficult to obtain a laminectomy that was wide enough because of the congenital narrowing of the spinal canal and the thickened laminae due to achondroplasia. Perioperative complications such as a dural tear are common during multilevel laminectomies in patients with achondroplasia [14]. Furthermore, decompressive surgery for ossification of the ligament carries an increased risk of dural injury [15]. In the present case, laminectomy for the severely stenotic spinal canal with adhesions of ossified ligamentum flavum was successfully completed with careful attention to avoid dural damage.

## Conclusion

In this report, we describe for the first time, to the best of our knowledge, a rare case of OLF in the congenital spinal stenosis associated with achondroplasia at the lumbar spine. The severe cauda equina symptoms were progressive, and laminectomy plus resection of the OLF resulted in acceptable improvement of the patient’s neurological symptoms.

## Consent

Written, informed consent was obtained from the patient for publication of this case report and any accompanying images. A copy of the written consent is available for review by the Editor-in-Chief of this journal.

## Abbreviations

OLF: Ossification of the ligamentum flavum.

## Competing interests

The authors declare that they have no competing interests.

## Authors’ contributions

KS prepared the manuscript and was involved in data analysis and the conception and design of the report. NM performed the surgery and helped to critically revise the manuscript. MH, YK and YI assisted in drafting the manuscript and reviewed the article. All authors read and approved the final manuscript.
